# Identifying the clinical indicator for surgical intervention in medication-related osteonecrosis of the jaw

**DOI:** 10.1007/s00774-025-01687-8

**Published:** 2026-01-22

**Authors:** Yoshiaki Tadokoro, Takumi Hasegawa, Daisuke Takeda, Junya Hirota, Kaito Uryu, Tatsuya Shirai, Yumi Muraki, Masaya Akashi

**Affiliations:** 1 Department of Oral and Maxillofacial Surgery, Kobe University Graduate School of Medicine, Kobe, 650-0017 Japan; 2 Division of Immunology, Department of Future Medical Sciences, Kobe University Graduate School of Medicine, Kobe, 650-0017 Japan

**Keywords:** Medication-related osteonecrosis of the jaw, Treatment strategy, Computed tomography, Demarcation line

## Abstract

**Introduction:**

Surgery has increasingly been reported as an effective treatment for medication-related osteonecrosis of the jaw (MRONJ), but concrete intervention criteria are lacking. On computed tomography (CT) images, the boundary between the healthy site and necrotic lesion, which we defined as “MRONJ demarcation line”, is sometimes visible. This study aimed to identify the factors associated with this boundary to improve surgical planning.

**Materials and methods:**

95 patients with MRONJ who underwent their first CT at our institution between May 2010 and June 2022 were included. The Mann–Whitney U test, Fisher’s exact test, and multivariate logistic regression analysis were performed. The cumulative incidence rates were calculated using the Kaplan–Meier method. Statistical significance was set at *p* < 0.05.

**Results:**

MRONJ demarcation line was observed in 63 patients and absent in 32. Significant associations were identified between MRONJ demarcation line formation and denosumab (*p* = 0.013), antiresorptive agent (ARA) discontinuation (*p* = 0.024), and periosteal reaction ( *p* = 0.034). The cumulative incidence rates of MRONJ demarcation line formation at 12, 24, and 36 months after discontinuation of high-dose ARA were 58.0%, 89.2%, and 96.4% for denosumab, and 29.9%, 68.8%, and 88.3% for bisphosphonates, respectively. In the low-dose group, the rates at 12, 24, and 36 months after discontinuation of denosumab were 41.7%, 51.4%, and 63.5%, respectively, while those for bisphosphonates were 22.2%, 35.8%, and 51.1%.

**Conclusion:**

Denosumab administration, ARA discontinuation, and periosteal reaction are significantly associated with the MRONJ demarcation line, which may help in establishing criteria for surgical intervention.

## Introduction

The initial reports of osteonecrosis of the jaw (ONJ) were published by Marx [1] in 2003, and since then, various types of antiresorptive agents (ARAs) have been reported to cause osteonecrosis [2]. ARAs, including denosumab and bisphosphonates, are commonly used to prevent skeletal-related events (SREs) in cancer patients and fractures in individuals with osteoporosis [3, 4]. The term medication-related osteonecrosis of the jaw (MRONJ) was defined by the American Association of Oral and Maxillofacial Surgeons (AAOMS) in 2014 [5]. The prevalence of MRONJ is estimated to be 0.02–0.3% in patients with osteoporosis and 0.5–5% in patients receiving oncological therapy [6–8].

Over time, the focus of MRONJ treatment objectives has shifted from palliative approaches to curative strategies, as evidenced by numerous studies demonstrating the success of surgical procedures [9, 10]. In 2022, the AAOMS emphasized that the primary goal is to cure MRONJ and enhance patient quality of life. They also noted that surgical options should be used to slow disease progression, with early surgical intervention potentially improving patient outcomes [2]. However, although there is considerable support for surgical intervention, the specific indications and procedures remain undefined. This is due to varying definitions of surgical treatment among researchers and the absence of detailed descriptions regarding surgical steps, such as the extent of bone resection and the timing of intervention. According to the recommendations of AAOMS, surgical interventions primarily consist of resection techniques aimed at removing necrotic and ischemic bone regions [2]. During the resection procedure, there exists a potential risk of excessive removal of healthy bone margins, and the success rate varies depending on the surgeon’s level of expertise [11]. Therefore, it is essential to establish clear and universal standards for surgical techniques.

**Fig. 1:**
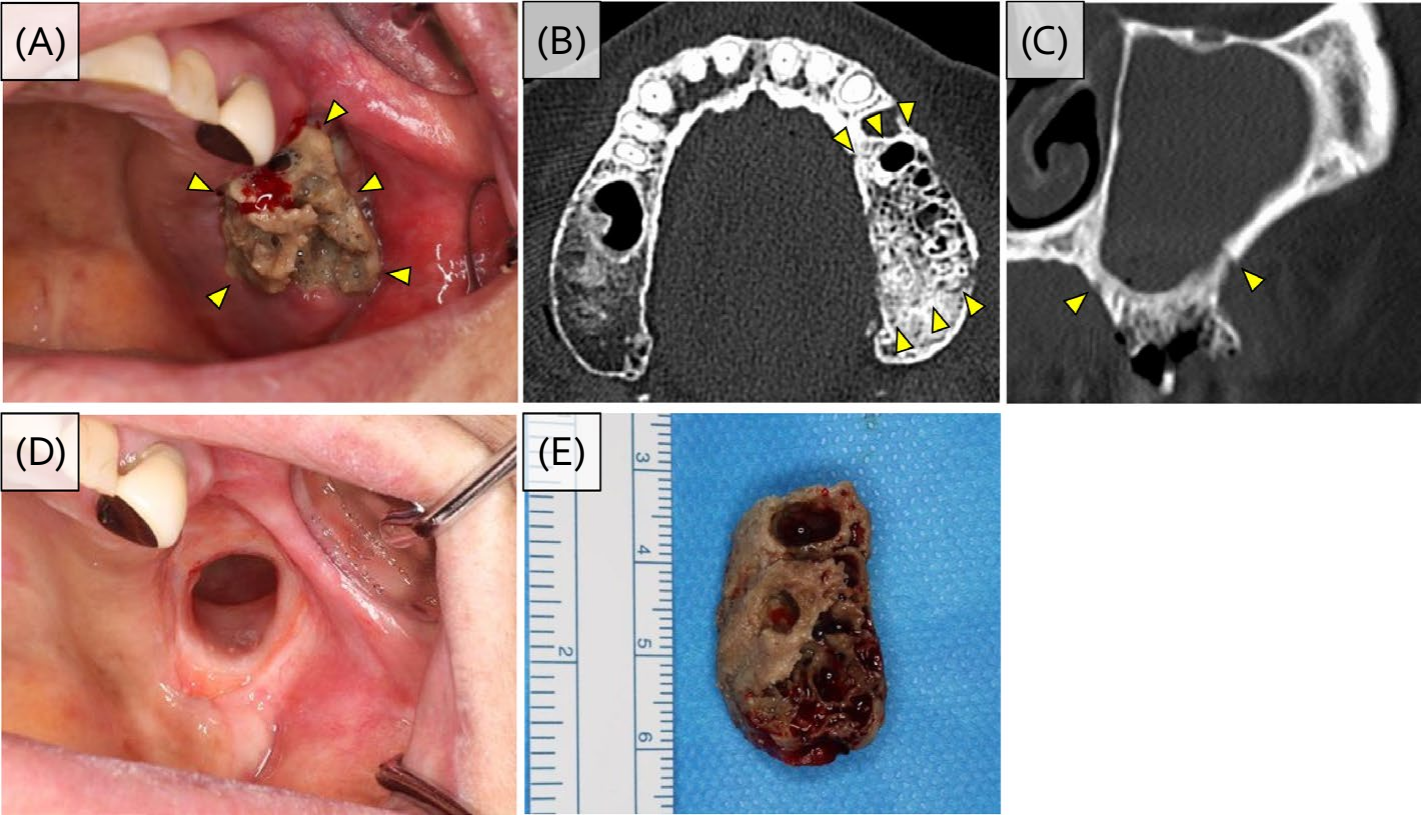
A patient with left maxillary MRONJ. **A** Intraoral photograph. Necrotic bone is exposed in the left maxilla (yellow arrowheads). **B** Axial CT image. A radiolucent thin line is visible in the osteosclerotic lesion, termed “MRONJ demarcation line” (yellow arrowheads). **C** Coronal CT image. MRONJ demarcation line is visible palatolingually. **D** Intraoral photograph taken 1 month after surgery. The wound is fully covered by the oral mucosa with the maxillary sinus opened. **E** Removed necrotic bone. *CT* computed tomography, *MRONJ* medication-related osteonecrosis of the jaw

Radiological assessments are essential for developing a treatment plan for MRONJ. Computed tomography (CT) enables accurate, periodic evaluation of the morphology and extent of the lesion [12]. Since ARA inhibit osteoclast formation and function and improve bone density, osteosclerosis is the most common feature of patients with MRONJ [13]. A periosteal reaction is observed in response to inflammation extending to the cortical bone, indicating new bone formation [14]. Sequestration is defined as calcified tissue completely separated from the adjacent viable bone [15]. Based on our experience, careful CT image analysis sometimes reveals a new visible line within the osteosclerotic lesion, which we defined as “MRONJ demarcation line” because it indicates the border between the MRONJ lesion and healthy bone (Fig. 1). This retrospective study aimed to analyze the relationship between various clinical findings and the MRONJ demarcation line, with the objective of refining criteria for surgical intervention.

## Materials and methods

### Patients

Patients diagnosed with MRONJ who underwent their first CT examination between May 2010 and June 2022 at the Department of Oral and Maxillofacial Surgery, Kobe University Graduate School of Medicine were included in this retrospective validation study. The MRONJ diagnosis and stage were determined based on the 2022 AAOMS guidelines [2]. After diagnosis, patients were followed periodically at our department, with serial CT scans performed to assess lesion extent and plan appropriate treatment. To rigorous evaluate the timing and emergence of the MRONJ demarcation line, patients were excluded if they had only a single CT scan or if a visible demarcation line was already present on their initial CT.

### Clinical data collection

The following clinical variables were collected from the electronic clinical charts: age, sex, follow-up period, stage, medical history, lesion site, onset trigger, the ARA type, dose, duration of ARA administration, ARA discontinuation, duration of ARA discontinuation, and antibiotic administration. Furthermore, chronic kidney disease, diabetes mellitus, and rheumatoid arthritis were recorded in the medical history data. The onset of MRONJ sites was recorded in the jaw region, including the maxilla or mandible, as well as the local dental region of the jaws, such as the incisors and molars, or both areas. The occurrence of tooth extraction and periodontitis in each case was recorded as a triggering factor of MRONJ. Patients treated with bisphosphonates and denosumab were assigned to the denosumab group, as MRONJ developed after switching from bisphosphonates to denosumab [16]. Patients receiving ARA for malignant diseases were classified as the high-dose group, and those treated for osteoporosis as the low-dose group, in accordance with internationally accepted criteria [17–19]. The duration of ARA therapy was defined as the period from initiation to the last administration. The term “ARA discontinuation” was used to collectively refer to temporary discontinuation, permanent cessation, or completion of ARA therapy. Discontinuation occurred either after completion of ARA therapy for the underlying disease or when the managing physician deemed withholding ARA feasible and prioritized MRONJ treatment over management of the underlying disease.

### CT image findings

**Fig. 2:**
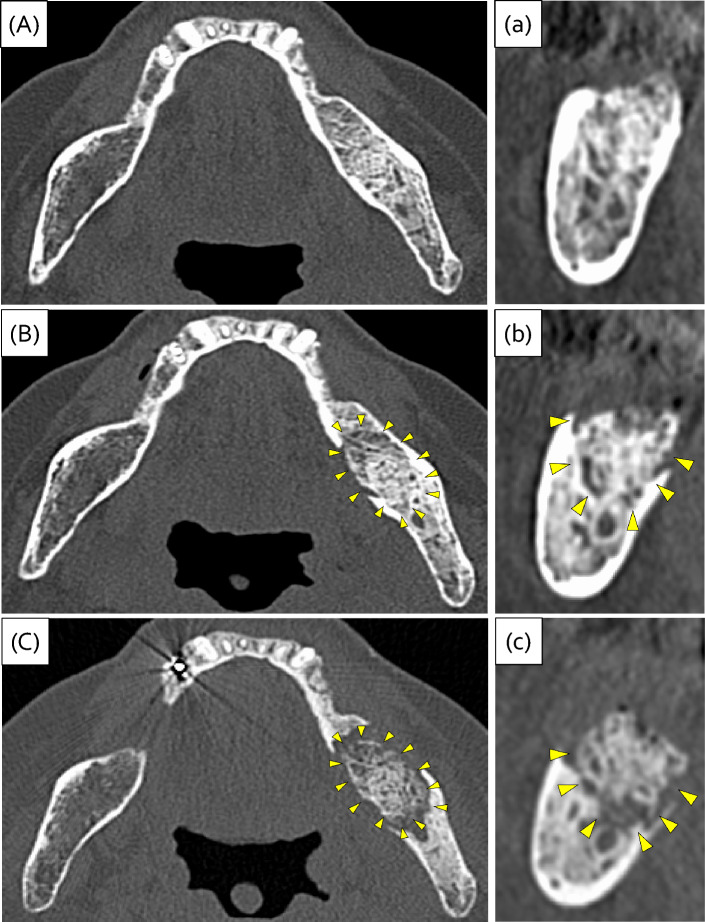
Temporal CT images of a patient with left mandibular MRONJ. **A** First axial CT image. Entire osteosclerosis is visible in the left mandible. **a** First coronal CT image. * mandibular canal. **B** Second axial CT image. A new area of mixed osteosclerosis and osteolysis 3 months after image (A). The visible line dividing this area from the osteosclerotic lesion is “MRONJ demarcation line” (yellow arrowheads). **b** Second coronal CT image. MRONJ demarcation line is the line between the lesion with incomplete sequestration and the osteosclerotic mandible. **C** Third axial CT image. The lesion consistent with MRONJ demarcation line is completely sequestrated from the left mandible 8 months after image (A). **c** Third coronal CT image. The boundary between the necrotic lesion and the osteosclerotic area is clear. *CT* computed tomography, *MRONJ* medication-related osteonecrosis of the jaw

CT scans were performed on a multi-detector CT scanner (Aquilion ONE or Aquilion Precision; Canon Medical Systems, Tokyo, Japan) with the following parameters: 120 kV tube voltage, 140–150 mA tube current, 0.5 mm slice thickness, 40 × 0.5 mm collimation, 0.5 s per rotation, 0.825 pitch, and FC 30 reconstruction kernel. Axial CT images of the jaws were acquired, and multiplanar reconstructions generated coronal and sagittal views. The presence of osteosclerosis, periosteal reaction, sequestration, and MRONJ demarcation line were evaluated on CT images. Based on clinical experience, we defined MRONJ demarcation line as a newly visible linear radiolucent boundary within osteosclerotic lesions, representing the interface between necrotic and viable bone (Figs. 1, 2). Each CT image was independently assessed by two observers (MA and YT). MA is a Fellow of the International Board for the Certification of Specialists in Oral and Maxillofacial Surgery, and YT is certified in oral and maxillofacial surgery.

### Statistical analyses

Statistical analyses were performed using GraphPad Prism 10 (GraphPad Software, La Jolla, CA, USA) and EZR software (Saitama Medical Center, Jichi Medical University, Saitama, Japan). The association between each variable and MRONJ demarcation line was analyzed using the Mann–Whitney U nonparametric test for ordinal variables and Fisher’s exact test for categorical variables. Statistical significance was set at *p* < 0.05. As osteosclerosis was observed in all patients, a univariate analysis could not be performed. The remaining variables were included in a multivariate logistic regression analysis. Osteosclerosis and sequestration were excluded from the multivariate analysis since their contingency tables did not include one or more categorical variables. Odds ratios (ORs) and confidence intervals (CIs) were also calculated. We conducted a Kaplan–Meier analysis to calculate the cumulative incidence rate of MRONJ demarcation line based on the ARA administration and discontinuation durations.

## Results

The study included 95 patients with MRONJ; MRONJ demarcation line was visible in 63 patients (66.3%). Table 1 presents the patient characteristics and univariate analysis results. The study included 32 male (33.7%) and 63 female (66.3%) patients; the mean age was 72.9 ± 10.5 (range, 47–92) years. Furthermore, 7 (7.4%), 62 (65.2%), and 26 (27.4%) patients had stages I-III MRONJ, respectively; MRONJ demarcation line was visible in 42.9%, 66.1%, and 73.1%, respectively (Table 1).

**Table 1:** Demographic, clinical characteristics, and CT findings in patients with or without MRONJ demarcation line

	MRONJ without demarcation line (−) (n = 32)	MRONJ with demarcation line ( +) (n = 63)	*p*-value
Age (years)			**0.526**
Range	53–92	47–91	
Mean (SD)	73.6 (9.7)	72.5 (10.9)	
Sex			**0.820**
Male	10	22	
Female	22	41	
Follow-up period			**0.738**
Range	2–137	2–79	
Mean (SD)	23.7 (25.7)	21.2 (18.4)	
Stages of MRONJ			**0.296**
I	4	3	
II	21	41	
III	7	19	
Onset area of MRONJ			**0.573**
Maxilla	7	10	
Mandible	25	53	
Local onset area in jaws			**0.061**
Incisor	2	0	
Molar	26	47	
Both	4	16	
Trigger factors of MRONJ onset			
Tooth extraction	9	29	**0.122**
Periodontal disease	10	22	**0.820**
Type of ARA			**0.018**
Denosumab	12	40	
Bisphosphonate	20	23	
ARA dose			**0.015**
High	13	43	
Low	19	20	
Duration of ARA administration			**0.621**
Range (months)	3–162	4–147	
Mean (SD)	42.1 (34.5)	46.6 (36.0)	
ARA discontinuation	24	62	** < 0.001**
Duration of ARA discontinuation			**0.030**
Range (months)	0–136	0–84	
Mean (SD)	16.8 (27.4)	17.5 (17.3)	
Antibiotics administration	19	52	**0.023**
Medical history			
Chronic kidney disease	2	8	**0.487**
Diabetes mellitus	6	10	**0.775**
Rheumatoid arthritis	5	7	**0.530**
CT image findings			
Osteosclerosis	32	63	NA
Periosteal reaction	9	35	**0.016**
0Sequestration	0	44	** < 0.001**

Fisher’s exact test was used to calculate *p*-values for categorical data, and the Mann–Whitney test for continuous data. A univariate analysis was not performed for osteosclerosis because it was present in all patients

*ARA* antiresorptive agent, *CT* computed tomography, *MRONJ* medication-related osteonecrosis of the jaw, *NA* not applicable

*p* < 0.05 is considered statistically significant

The presence of MRONJ demarcation line did not differ based on age, sex, follow-up period, stage, onset jaw or region, onset trigger, duration of ARA administration, or medical history including chronic kidney disease, diabetes mellitus, and rheumatoid arthritis (Table 1). However, the presence of MRONJ demarcation line significantly differed based on the ARA type. Denosumab and bisphosphonates were administered to 52 (54.7%) and 43 (45.3%) patients, respectively (Table 1). Denosumab use was a significant factor in the formation of MRONJ demarcation line (*p* = 0.018) (Table 1).

The ARA dose was also significantly associated with the demarcation line. Overall, 56 (58.9%) patients received high-dose ARA to treat metastatic bone cancer, whereas 39 (41.1%) patients received low-dose ARA for osteoporosis treatment; MRONJ demarcation line was significantly more common in the high-dose group than in the low-dose group (*p* = 0.015) (Table 1). Moreover, 86 patients (90.5%) discontinued ARA during the follow-up period (Table 1). Of the 9 patients (9.5%) who continued ARA, 3 (33.3%) underwent cancer treatment, and 6 (66.7%) were treated for osteoporosis. ARA discontinuation was also significantly associated with the MRONJ demarcation line in the univariate analysis (*p* < 0.001) (Table 1).

In this study, patients underwent wound cleaning with or without antibiotic administration as a conservative treatment. Antibiotic administration was significantly associated with MRONJ demarcation line (*p* = 0.023) (Table 1).

**Fig. 3:**
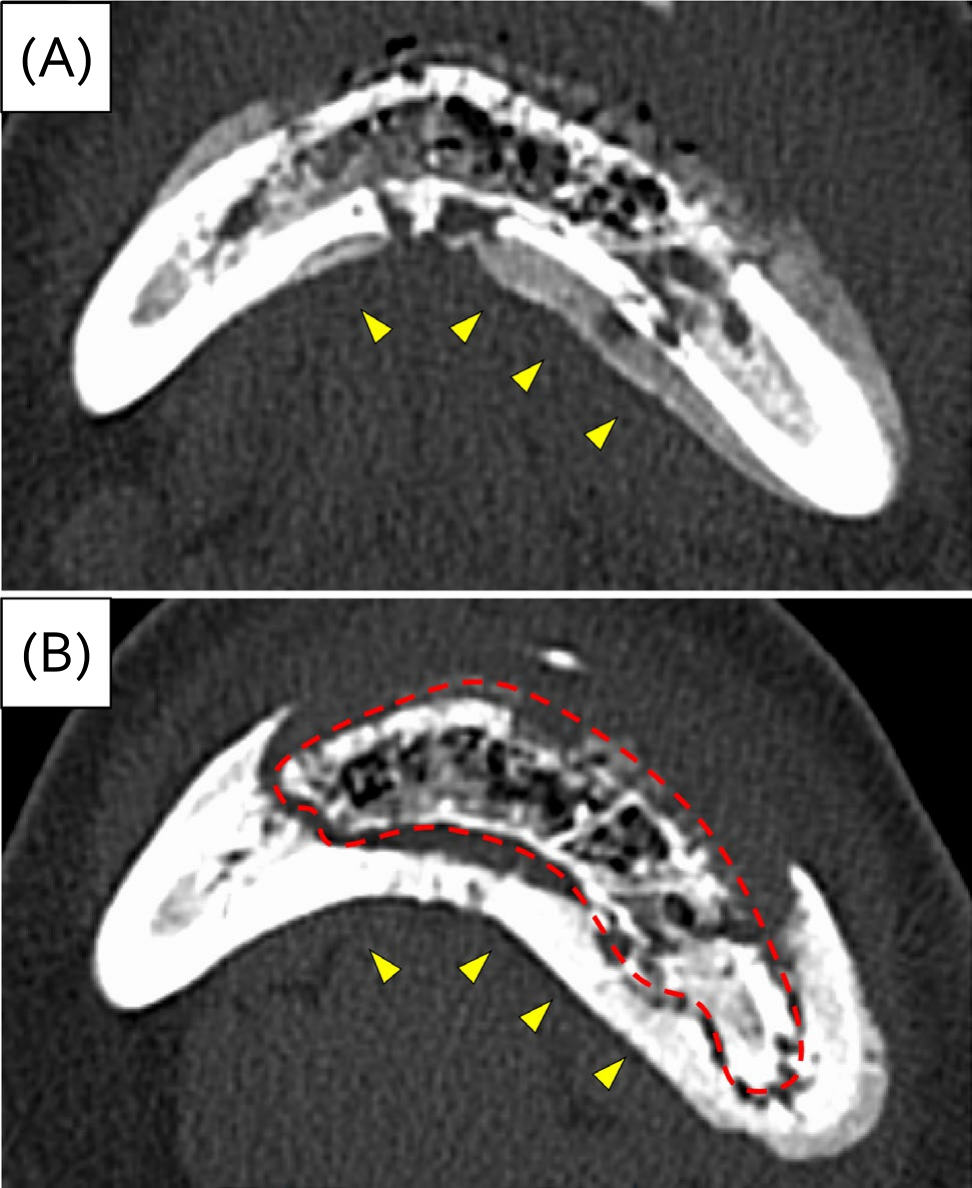
Temporal CT images of a patient with mandibular MRONJ. **A** First axial CT image. A discontinuous periosteal reaction is observed on the lingual side (yellow arrowheads). **B** Second axial CT image. 12 months after image (A), the lingual side of the periosteum is developed, and new bone formation is observed along the area of the periosteal reaction. The necrotic lesion appears as the region separated by MRONJ demarcation line (red dotted line)

Osteosclerosis, periosteal reaction, and sequestration were observed on the CT images of 95 (100%), 44 (46.3%), and 44 (46.3%) patients, respectively (Table 1). Among the 63 patients with a visible MRONJ demarcation line, 63 (100.0%), 35 (55.6%), and 44 (69.8%) had osteosclerosis, periosteal reaction, and sequestration, respectively (Table 1). Periosteal reaction was observed either prior to or concurrently with the appearance of MRONJ demarcation line (Fig. 3). Periosteal reaction and sequestration were significant predictive factors for MRONJ demarcation line in the univariate analysis (*p* = 0.016 and *p* < 0.001, respectively) (Table 1).

Multivariate analysis revealed that the following factors were significantly associated with MRONJ demarcation line: denosumab (OR, 3.55; 95% CI, 1.30–9.67; *p* = 0.013), ARA discontinuation (OR, 13.40; 95% CI, 1.40–129.00; *p* = 0.024), and periosteal reaction (OR, 3.01; 95% CI, 1.09–8.31; *p* = 0.034) (Table 2).

**Table 2:** Results of multivariate analysis between variables and MRONJ demarcation line

	95% CI	OR	*p*-value
Denosumab	1.30–9.67	3.55	0.013
ARA Discontinuation	1.40–129.00	13.40	0.024
Periosteal reaction	1.09–8.31	3.01	0.034

*OR* odds ratio, *CI* confidence interval

*p* < 0.05 is considered statistically significant

**Fig. 4:**
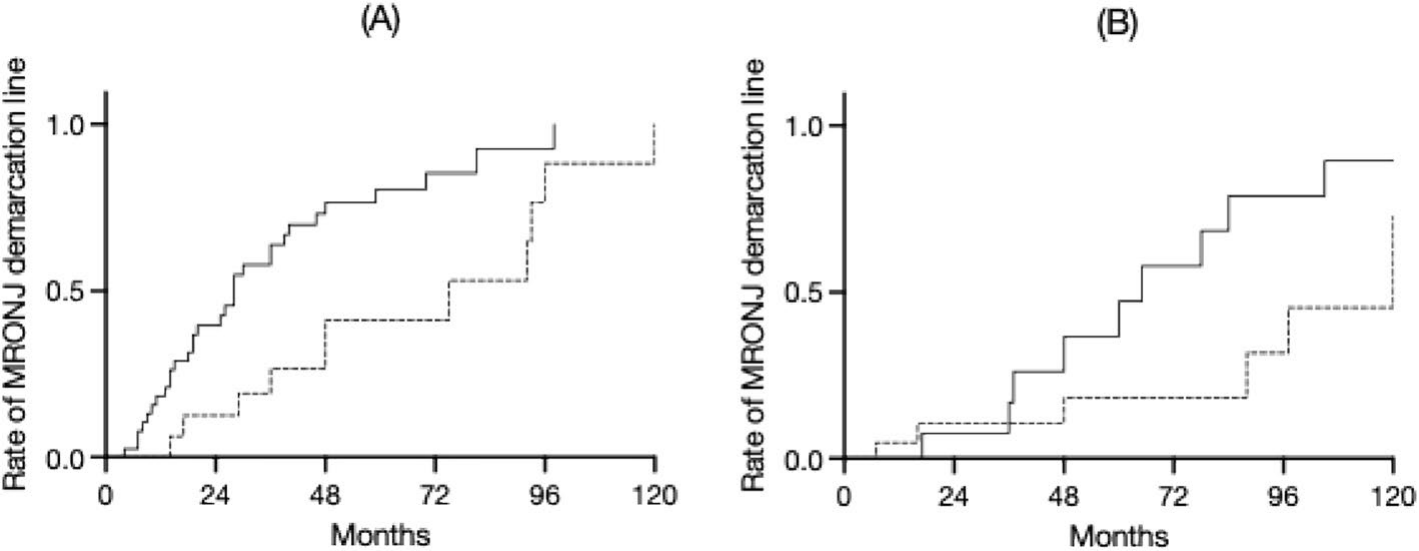
Cumulative incidence rates of MRONJ demarcation line based on the duration of ARA administration. The rates for patients administered **A** high-dose and **B** low-dose ARA.ARA, antiresorptive agent; MRONJ, medication-related osteonecrosis of the jaw; solid line, denosumab; dashed line, bisphosphonates

**Fig. 5:**
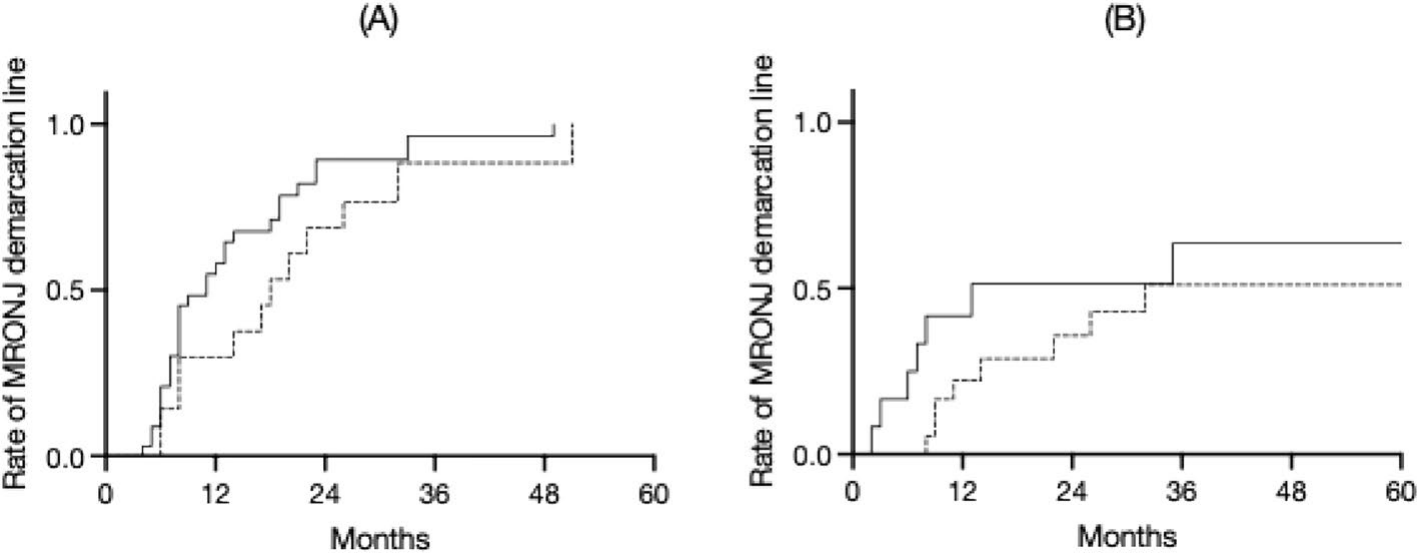
Cumulative incidence rates of the MRONJ demarcation line based on the duration of ARA discontinuation. The rates for patients administered **A **high-dose and **B** low-dose ARAs.ARA, antiresorptive agent; MRONJ, medication-related osteonecrosis of the jaw; solid line, denosumab; dashed line, bisphosphonates

Figure 4 shows the relationships between the cumulative incidence rates of MRONJ demarcation line and ARA administration duration. The MRONJ demarcation line incidence rates 36, 48, and 60 months after initiating high-dose ARA were 63.7%, 76.5%, and 80.4% for denosumab, and 26.6%, 41.3%, and 41.3% for bisphosphonates, respectively (Fig. 4A). The incidence rates 36, 48, and 60 months after initiating low-dose ARA were 16.9%, 36.7%, and 47.3% for denosumab, and 10.7%, 18.2%, and 18.2%, respectively (Fig. 4B). Figure 5 presents the cumulative incidence rates of MRONJ demarcation line based on the duration of ARA discontinuation. The MRONJ demarcation line incidence rates 12, 24, and 36 months after discontinuing high-dose ARA were 58.0%, 89.2%, and 96.4% for denosumab, and 29.9%, 68.8%, and 88.3% for bisphosphonates, respectively (Fig. 5A). The incidence rates 12, 24, and 36 months after discontinuing low-dose ARA were 41.7%, 51.4%, and 63.5% for denosumab, and 22.2%, 35.8%, and 51.1% for bisphosphonates, respectively (Fig. 5B).

## Discussion

In the present study, ARA discontinuation, denosumab, and periosteal reaction were identified as independent predictors of MRONJ demarcation line. To the best of our knowledge, predictors of well-defined radiographic boundary of the lesion have not been previously reported. Kojima et al. reported a low cure rate in non-osteolytic MRONJ, possibly attributable to the difficulty in accurately determining the extent of surgery [16]. The most recent Italian position paper recommends perioperative ARA discontinuation and advocates dose- and agent-specific surgical management [18]. Herein, we propose practical surgical strategies focusing on MRONJ demarcation line formation according to the type and dose of the agent, with consideration of ARA discontinuation.

Although a 3-month ARA discontinuation has been described as unnecessary for surgical planning, our findings indicate that long-term discontinuation significantly contributes to clearer surgical margin delineation in MRONJ [20]. In patients receiving high-dose ARA, the feasibility of ARA discontinuation should be carefully discussed with the attending physician, as these agents are often prescribed for bone metastatic cancer. Given that the cumulative incidence rates of MRONJ demarcation line exceed 50%, we recommend discontinuation of denosumab for 12 months and bisphosphonates for 24 months in patients eligible for both long-term ARA discontinuation and surgery. In patients treated with low-dose ARA, discontinuation of 24 months for denosumab and 36 months for bisphosphonates may be useful for developing surgical treatment plans. Several studies reported that denosumab cessation can lead to a rebound effect, increasing the risk of multiple vertebral fractures [21, 22]. Bisphosphonates show a long-lasting inhibitory effect on bone remodeling, but their duration after discontinuation has not been fully clarified. Herein, we recommend switching these agents to alternative medications in MRONJ patients with osteoporosis who are scheduled for surgery.

Periosteal reaction was observed as part of the healing process (Fig. 3). Previous studies have described periosteal reaction as a risk factor for treatment failure due to bacterial infection [23, 24]. None of the patients with periosteal reaction were cured by conservative treatment, indicating that surgical intervention may be the only effective option [14]. Taken together, surgical intervention should be considered when MRONJ demarcation line becomes apparent in patients presenting with periosteal reaction. Periosteal preservation may promote bone regeneration when infection control is successfully achieved. However, because the extent of new bone formation remains uncertain, careful consideration should be given to the potential risk of pathological fracture when determining the extent of periosteal and newly formed bone preservation.

Because most patients with MRONJ are elderly or have cancer-related systemic conditions, it is essential to minimize surgical invasiveness and clarify the appropriate timing of surgical intervention. Our approach, which enables more accurate estimation of the extent of resection, has the potential to improve both esthetic and functional outcomes. Furthermore, establishing clear criteria for surgical timing may facilitate more precise and individualized treatment planning. The ability to accurately define the minimal necessary surgical area would therefore represent a major clinical advantage. The present study suggests that this goal may be achievable, providing a useful step toward more individualized surgical management of MRONJ. Future large-scale studies should address the occurrence of MRONJ demarcation line with continued ARA administration.

In conclusion, this study identified associations between denosumab, ARA discontinuation, and periosteal reaction and the formation of MRONJ demarcation line. Our data strongly supports the efficacy of ARA discontinuation for managing patients with MRONJ when planning surgical interventions, a benefit that has not been previously revealed.

## Data Availability

The data generated in this study are available upon request from the Corresponding Author.
